# It Happened in the Desert, in Libya and in Italy: Physical and Sexual Violence Experienced by Female Nigerian Victims of Trafficking in Italy

**DOI:** 10.3390/ijerph20054309

**Published:** 2023-02-28

**Authors:** Sarah Adeyinka, Ine Lietaert, Ilse Derluyn

**Affiliations:** Department of Social Work and Social Pedagogy, Ghent University, 9000 Ghent, Belgium

**Keywords:** sexual violence, physical brutality, human trafficking, smuggling, Nigerian, Italy

## Abstract

Nigerian girls and women constitute a large percentage of African victims of human trafficking in Italy. Extensive research has been conducted on the causes, push-and-pull factors, and the perpetrators in the phenomenon of trafficking Nigerian women and girls into Italy. However, limited data exist on the women and girls’ narratives of their experiences during their migratory journey from Nigeria to Europe. Using data collected through a mixed method, longitudinal design, 31 female Nigerian victims of trafficking in Italy were interviewed for this study. This study gives voice to the experiences of sexual violence that these women and girls encounter during transit, leading to many of them arriving in Italy severely traumatized. It also discusses the health impact of these experiences and the different survival strategies that they are forced to employ. The study shows how sexual and physical violence is employed by smugglers, traffickers, and people in authority alike. It shows that the violence experienced along the way does not end after arrival in the destination country (in this case, Italy), but is, in some cases, exacerbated and similar to previous experiences of violence.

## 1. Introduction

In 2022, an estimated 50 million people were enslaved globally [1], and women and girls account for more than half of trafficking victims [2]. Although European countries record the highest number of conviction rates of traffickers, they also have the highest number of trafficked victims from West Africa, who make up 9% of identified victims in the region [2]. Between 2017 and 2018, “*majority of all detected Nigerian trafficking victims in the EU were exploited in the prostitution industry*” [3] (p. 16) and Nigerians remain one of the top five countries of origin of non-European victims of trafficking in the European Union [4]. Nigerians also constitute a large percentage of refugees and migrants arriving in Europe, accounting for 23% of all land and sea arrivals between 2017 and mid-2020 [3]. For over a decade, Nigerian nationals in Italy constituted the highest number of victims of human trafficking that end up in the care of social protection services [5].

Sexual violence is defined by the World Health Organization (WHO) as “*any sexual act… or acts to traffic, or otherwise directed, against a person’s sexuality using coercion, by any person regardless of their relationship to the victim, in any setting, including but not limited to home and work*” [6] (p. 149). Sexual violence is inevitably part of the experiences of victims who are trafficked for sexual exploitation purposes [7]. Additionally, studies have documented how refugee women experience extensive violence en route to the country of destination, including sexual violence [8,9,10,11].

Some studies also document how refugee women also become victims of violence and abuse after arrival in the destination country, for example, in asylum centers or refugee camps [12,13]. When referring to violence, we use the definition adopted by the World Health Organization (WHO):

*The intentional use of physical force or power, threatened or actual, against oneself, another person, or against a group or community, that either results in or has a likelihood of resulting in injury, death, psychological harm, maldevelopment or deprivation*.

[6] (p. 5)

Many refugees and migrants traveling irregularly experience emotional and socio-economic violence during their migration trajectories [10]. However, female refugees and women and girls migrating irregularly are more likely to experience sexual violence during their trajectories [9,11], in transit locations [8], and even in the country of destination [12]. Research shows that sexual violence has complex and lasting mental and physical health effects on victims [14], especially anxiety, depression, and post-traumatic stress [15] which may be exacerbated in the case of repeated experiences of (sexual) violence and abuse [16,17]. As articulated by Peltola [18] (p. 4), “*arguably, rape is the most violent, humiliating, and brutal offense inflicted on a woman… (it) serves as a daily reminder of the brutality of war. Rape leaves scars, physical, emotional, and psychological*”. We also know that rape and sexual violence are often used for the portrayal of power and dominance [19] and to instill fear by maiming victims through acts of cruelty [20]. Gaventa [21] posits that powerlessness is not a personal problem of the powerless, but rather a social circumstance that is embedded in inequality and the deprivation of social solutions, validating the importance of understanding ‘context, time and milieu’ when studying violence [22]. This is important in our study, which illustrates the continuation of violence in different locations and contexts, and how circumstances may increase vulnerability.

Commendable research has been conducted on the trafficking of Nigerian women and narratives of the Nigerian women in detention centers in Libya and Italy. These studies show the arduous and complex journeys that they undertake to get to Europe, and the exploitation that happens along the way [23,24]. They highlight these women and girls’ experiences of psychological, physical, and sexual violence, including forced abortions without the administration of anesthesia [25]. Research also shows the uncertainty that they experience after arrival in the destination country, how they attempt to craft a new life for themselves [26], and how their experiences are reflected upon by Nigerian cultural mediators [27].

Yet, little is still known about to what extent the experiences of violence, especially sexual violence, and related exploitation are part of the migration trajectories of female victims of trafficking before they arrive in the country of destination. Additionally, it remains unclear whether this group is also vulnerable to (sexual) violence once they have left the trafficking networks.

This study focuses on the experiences of sexual violence, as relayed by Nigerian teenage and adult victims of human trafficking for sexual exploitation who arrived in Italy irregularly via the central Mediterranean route. It discusses the violence perpetrated against them by various actors throughout their trajectories, and how the violence continues after arrival in their destination country, Italy. To show that the violence is perpetrated not just in the migrants’ countries of origin only by members of organized crime, we discuss the experiences of violence (predominantly, sexual violence) in two transit countries (Niger and Libya) and after arrival in Italy. We also discuss the perpetrators, who include members of organized criminal networks and armed militia, soldiers, local men, and caregivers. Research shows that “*when certain situations or events exceed the individual’s own capacities, general self-efficacy decreases, and this may play an important role in stress generation*” [28] (p. 2). This is seen in the different mechanisms employed by the participants and how they are forced to make choices that may not always end their pain but, at least, lessen it, hereby contributing to the literature on migrants’ trajectories, which show a continuous process of negotiations and disruptions [29,30].

## 2. Materials and Methods

### 2.1. Procedure

The European-Research-Council-funded ChildMove Project, which this study is part of, is a longitudinal study with a mixed methods design. The project documents the impact of flight experiences on the psychosocial wellbeing of unaccompanied young refugees and migrants during their migration trajectories. The study used a two-year time frame, during which the participants were repeatedly interviewed along their migration journeys.

This study followed 31 Nigerian teenagers and young women who arrived in Italy between January 2016 and June 2018 over a period of two years in three measurement moments (M1, M2, and M3). Three participants were contacted and recruited for the study based on the researcher’s (first author) communication with them, stemming from her previous work in Italy, while 28 women were recruited from three Non-Governmental Organizations (NGOs) in the regions of Campagna, Piemonte, and Sicily in Italy. These organizations had at least one of their main focuses on supporting victims of human trafficking and had living homes or apartments where the women and girls lived. Although one participant was in search of housing and squatting with friends during the first interview, the remaining 30 participants lived in the NGO-provided/supported accommodation during the time of the first interview.

While the participants were all victims of trafficking, their experiences of sexual exploitation by traffickers began at different parts of the journey. For some, the trafficking for sexual exploitation began during the journey in Libya where they were forced into prostitution; for others, it continued after arrival in Italy; and for a third group, Italy is where they were first forced into prostitution. There were a few participants, however, whom, upon arrival in Italy, were referred by NGOs to the relevant authorities as victims of trafficking; therefore, their sexual exploitation at the hands of traffickers did not continue after arrival in Italy.

The data collection consisted of self-reporting questionnaires and semi-structured interviews (cf. Section 2.3). Prior to the field work, the researcher had a list of credible organizations as part of a referral network to whom each participant could be referred to if needed, with their consent. The participants were in specific safe houses under the care of NGOs where they did have regular mental health support. The researcher asked participants during each interview if they wanted/needed mental and/or physical health support, and some said yes. Those who said yes consented to her contacting the director of the shelter directly on their behalf, which she did. Those mechanisms were essential for us to conduct this study in an ethical manner.

Before each in-depth interview, the researcher explained the study to each participant and requested her informed consent. In some cases, she was able to explain the research to the participants first in groups and then individually, to ensure that they understood the goal of the study and that they were free to participate, without any consequences. Being Nigerian herself and a native English, Yoruba, and Pidgin English speaker, the first author conducted all the interviews without an interpreter and fluctuated between languages based on which language(s) each participant was most comfortable with. The interviews were fully transcribed and translated into English before data analysis and coding began. Being a fellow Nigerian woman endeared the researcher to the participants very quickly and enabled her to gain access to them. She was considered as and referred to as either ‘sister’ or ‘aunty’, as is common practice in Nigeria because she was older than the participants. This role that she was given, however, came with its own challenges because as much as she emphasized her role as a researcher, some participants saw her as their kin. Therefore, several conversations on ethics and positionality ensued between the researcher and the co-authors throughout the duration of the study.

### 2.2. Participants

Thirty-one female Nigerians were recruited for this study, ten teenagers and twenty-one adults. Briefly, 10 were 16 or 17 years old; 16 were between 18 and 25; and another 5 participants were between 26 and 35 years old.

While 32 participants were approached, 31 participants agreed to participate in the study and one participant declined participation because she did not want to discuss her past. Most of the participants were from Edo (n = 20) and Delta (n = 6) states, while the remaining (n = 5) women were from other states in Nigeria. Most participants indicated that they were pushed to travel for economic reasons and travelling overseas seemed to be the logical step after exhausting other options to earn more income in Nigeria; however, they all ended up being trafficked for sexual exploitation.

All participants had been out of the trafficking networks for a minimum of six months when the first set of interviews was conducted. We intentionally interviewed participants who had been out of the networks for that duration because they would have received some (psychological and social) assistance, were no longer under the control of networks, and were in relatively stable environments where support was available. Thirty-one participants completed the first measurement moment, twenty-two participated in two measurement moments, and sixteen completed all three. The participants who did not continue the study had relocated from Italy, had changed their phone numbers and could not be reached, or chose not to continue because they did not want to revisit the painful memories.

Ethical approval was requested from and granted by both the Commissione per L’Etica della Ricerca e la Biotica, (Research Ethics and Integrity Committee) Italy and the Ethics Committee of the Faculty of Psychology and Educational Sciences at Ghent University, Belgium.

#### Data Collection and Analysis

The data collection consisted of self-reporting questionnaires and semi-structured interviews. For this study, we mainly relied on qualitative data, because of the rich insights these data provide into participants’ experiences of violence during their migratory journeys. However, Behrendt et al.; Orsini et al.; Pfeiffer et al. [31,32,33] provide in-depth information on the quantitative aspect of the project, which this study does not delve into.

### 2.3. Qualitative Measures

The data were collected using semi-structured interviews, which included questions on participants’ demographic information, migration experiences, experiences of violence along the way, living conditions, daily stressors, coping strategies, support networks, and future aspirations. During follow-up interviews, the questions focused on what changed between the last interview and the ongoing one. The average time that the in-depth interviews lasted for was 50 min; however, some interviews lasted over two hours. The duration depended on how much the participants were willing to share, and how much time they had available for the interview.

Since violence and witnessed and/or experienced violence were predominant in the narratives of our respondents, we further explored that theme during the analysis using thematic analysis and composite narrative.

The data analysis was conducted using NViVo (data analysis software), first, to gain an overview of the data and to better understand, capture, and interpret information in the data that may have otherwise been overlooked [34]. Utilizing a composite narrative also allowed for a clearer presentation of the participants’ experiences with an increased understanding of the (prevalent) theme [35].

Next, the data were coded in NViVo for patterns because “*patterns suggest a multiplicity of elements gathered into the unity of a particular arrangement*” [36] (p. 136). Patterns also demonstrate importance and habits in people’s daily lives [37]. This was particularly important in our study, as the patterns found during data analysis included the use of violence, especially sexual violence. Violence was then a top-level code, which we coded further to discover the type(s) of violence, location of violence, and (if known) the perpetrator(s) of violence.

The next top-level code was any mention of coping mechanisms and survival strategies to better understand how the participants coped with their experiences of violence. This led to the last top-level code, which was the impact of the experiences on the participants’ physical, emotional, and psychological wellbeing.

## 3. Results

When each participant was asked to describe her journey from Nigeria to Italy, the theme of violence was overwhelmingly present; among the different forms of violence, sexual violence and other forms of physical brutality were predominant.

**Figure 1:**
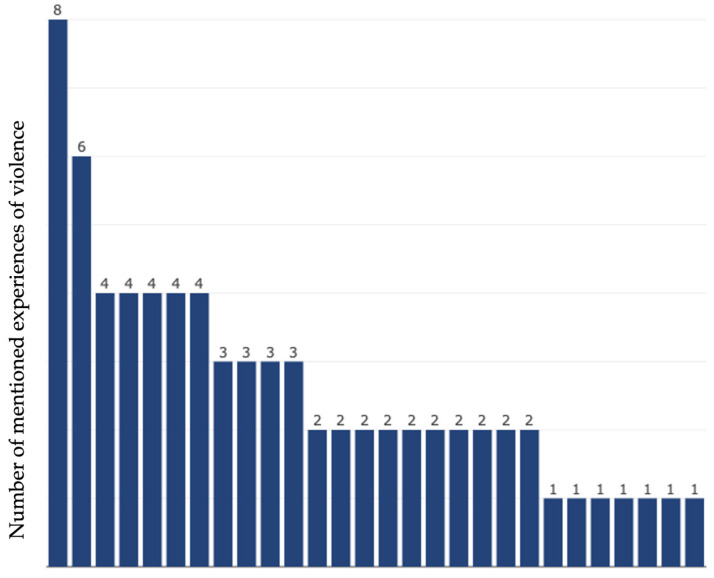
Participant’s mention of experiences of violence (each bar representing one participant).

This section, therefore, presents the participants’ experiences of physical and sexual violence and where they had those experiences during their migration trajectories. It shows how the violence continues from Niger, being the first country of transit, into Libya, and how it continues after arrival in Italy. Of the 31 participants interviewed, 28 personally experienced rape and/or other forms of sexual violence, and data from the coding shows how many times each participant mentioned their experiences of violence during the interview (see Figure 1 below).

Rape and sexual assault were especially common in the participants’ narratives. Some of the participants were numb and almost dissociated while telling their stories, some were visibly angry and expressive, while others wept. For many of the participants, the violent experiences began as soon as they embarked on the journey. It began with the juju rituals, then continued more intensely after departure from Nigeria in the neighboring country of the Republic of Niger, where the perpetrators were mainly smugglers, nomads, and armed militia in the desert. In Libya, the perpetrators were smugglers, traffickers, armed militia, armed officers, and prostitution clients. The violence continued after arrival in Italy, and the perpetrators were mainly traffickers, clients and pretend clients, locals, and, in a few cases, staff of non-governmental organizations, after the participants had left the trafficking networks. Owing to the traumatic experiences that these teenagers and women experience during transit and after arrival in Italy and its extensive health impact, their narratives show how the participants were forced to adapt and focus on ways to ensure their survival despite the traumatic situations that they encountered.

### 3.1. Experiences of Violence during the Migration Trajectories

All participants departed Nigeria with the belief that the situation overseas would be better, that they would be able to earn more money, and that the conditions of their family members back home would drastically improve because of their overseas migration.

*I left Nigeria in search of a better life and a way to help take care of my younger ones since my dad is no longer alive to take care of us. Dad died, then mum remarried, and my stepfather hates her. He will beat me and my younger ones and sometimes chase me out of the house and not let me come back for days. Even then, my mother would just cry, she did not do anything to stop him or even try to help me. So, I realized that no one will help me raise my siblings… they are my younger ones, who will take care of them, if not me? It was while I was hustling and looking for a way to make enough money that I met a woman who promised me a job in Libya as a stylist*.

*(Adult, M1)*

Several participants had no doubt that their journey would be beneficial to their families and, therefore, left their children at home in the care of family members. These young women left their children back home with the hopes of each woman sending for her child in a few months after arriving in Europe and saving up enough money.

*Once I get a good job, it will be perfect because I will be able to support those at home, especially my mother who is the one taking care of my son that I had to leave at home when I came. Then I can bring him here and we will be together.*

*(Adult, M1)*

*So, I gave my 7-year-old child to my mother and told her not to tell anyone about my journey.*

*(Adult, M1)*

That, however, did not happen and was one of their biggest regrets and disappointments, since none of the participants over the course of our study were reunited with their children in Nigeria.

*My son is with my mother [in Nigeria] and we talk on the phone a lot, he is getting so big, and he is the reason why I am still alive and hustling to survive.*

*(Adult, M2)*

One form of violence that they experienced, but never referred to as violence, was the juju rituals that they underwent in Nigeria, and for one participant in Libya. In the Nigerian human trafficking networks, before embarking on the journey to Europe, the women and teenagers are taken to a shrine where they swear an oath to conceal the identity of the trafficker and to repay the amount determined by the trafficker as the cost of the journey [38,39,40].

*The woman took me to the shrine to swear that I would repay her for the journey; they took my picture, gave me kolanut and something else to eat, and then I swore.*

*(Adult, M1)*

Personal items, such as hair, nails, underwear, and even blood, are then taken from them to hold them to their vows, and they are told that if they break their vows, evil will befall them and their loved ones [25,41,42]. While the participants described the rituals with disgust, solemnity, and even sadness, none referred to them as violent or brutal. It was almost as if they were not considered violence because they were not beaten or raped and, thus, considered as a spiritual covenant alone, not an example of brutality [43].

#### 3.1.1. Experiences of Violence: Republic of Niger

The participants’ narratives on violence began as soon as they left Nigerian soil and arrived in Niger, where more than half of the participants (n = 16) indicated that they experienced and/or witnessed physical and/or sexual violence during transit. The violence in Niger mainly happened in the Sahara Desert, except for one account of experienced violence in a small village where a participant had to wait.

*The desert (in Niger) was hell and Libya too. In the desert, it was very hot and dry, you just see sand for days without end. No water, you drink your own pee or if someone else has more, they can share their pee with you- whether you are on your period, you drink. There were so many dead bodies and skeletons in the desert, dry bones, people’s clothes, that place is hell.*

*(Minor, M1)*

Survival meant doing what they deemed disgusting, and, for many participants, the desert was where they saw a corpse for the first time in their lives. It was also where they witnessed the death of other migrants and realized that a successful journey was not guaranteed.

*The desert was horrible; heavy sun and many people died there. In the car behind our own, one boy died. The boy died because of too much sun and no water... The desert was the most difficult part of the journey- no beginning, no end.*

*(Minor, M1)*

The Sahara Desert was where many participants experienced sexual violence and physical brutality by different actors for the first time on the journey.

*We jumped on the Hilux that took us to Agadez and the desert. That was the first time they forced me to have sex, the guys at the desert checkpoint, it was very, very bad. I don’t want to talk about it. The desert was scary, thirsty, windy, and heat, all together.*

*(Minor, M1)*

*In the desert, I was beaten and stripped by the Niger guys who wear turbans that they wrap around their head and face. The men put their hands on my body and inside it claiming to look for money and hidden things. I was touched anyhow and humiliated.*

*(Adult, M1)*

Niger was also the first place where they witnessed violence against their fellow travelers:

*In beginning, the drivers were from Niger, they were bad and tried to sleep with as many girls as possible; then the Arab guys took over, they were heartless- they beat up and slept with us. They would yell ‘Aiya’- meaning ‘let’s go’ once they were ready to move after we stopped in the desert, and anybody that was not ready would be left behind. We were 38 in the back of the Hilux [truck] and one time, one of the girls fainted… Some soldiers met us there in the desert and they helped her. Although some of the soldiers were also bad ones who were shooting at us for fun, they took all our money and phone and they slept with us again. We were in the desert for five days… We even drank water from one well that had dead bodies inside, it was just terrible. *

*(Adult, M1)*

This was also where many participants lost faith and the hope of being rescued from the dangers of the journey. The participants often referred to Arabic-speaking men as Arabs or ‘Arabu’, and it was not always clear if those men were from Niger, Libya, or both countries. The experienced exploitation by those who were supposed to lead them to safety and soldiers who could have protected them marked the beginning of their disillusionment in the journey, perhaps solidifying a distrust for people with power.

*I just found out that I was pregnant before the journey, and I was very sick and afraid. Then one of the smugglers tried to force me in the desert. So, one guy tried to save me, but the smuggler shot him and killed him. So, I had no choice but to give in; the man raped me, and I also saw him killing the person that tried to help me.*

*(Minor, M1)*

In addition to the trauma and pain that accompanies rape, this teenager also witnessed the murder of a fellow traveler who tried to save her. Although this was not discussed in the interview, she may also be tormented by feelings of guilt that she was responsible for the loss of someone else’s life and might be blaming herself for not giving in earlier. This experience may also prevent the participant from disclosing her experiences to receive the support and health care that she needs.

#### 3.1.2. Experiences of Violence: Libya

In Libya, witnessing and experiencing violence continued for many participants, and the locations were broadened. It happened in detention centers and prisons (under the control of Libyan authorities or armed gangs), in the ghettos and brothels (while under the control of traffickers), at the seaside (under the control of traffickers), and in other locations, which include the desert and unknown locations in the outskirts. While Libya was also a transit country, unlike Niger, where they experienced violence and continued their journey shortly afterwards, the violence in Libya was continuous, as the average number of months spent there by the 31 participants was 19.91 months. During that time, they were under the control of traffickers or smugglers. Some of the participants ended up in detention, some were abused and forced into prostitution, but they were all waiting to be told that they could finally leave for Italy.

- Detention

Many participants were detained in Libya, either by the Libyan authorities or the violent gang referred to as ‘Asma boys’. Another study [44] carried out among migrants who travelled to Europe through Libya, also mentions ‘Asma boys’ and refers to them as armed street gangs that extort migrants. Regardless of who the captors were, violence and abuse were continuously meted out to the participants.

*Yes, my life was in danger back there [Libya], also because many girls died while working there… Some were due to punishment. There were some that wanted to escape and maybe they were caught in the act of escaping so they will be like taken away and beaten or so.*

*(Adult, M1)*

The normalcy with which this participant talked about other migrants being beaten to death signifies a level of numbness that may be in place to protect her from the horrors of what she saw and experienced, and verifying the torture and brutality that all the participants agreed was the norm in Libya.

Yes, the boy’s name is ***. He was shot with a gun. That’s not even mentioning that they used to beat us up a lot. They would complain that we were making noise and start beating us up. They were so wicked. There were two of them guarding us. One of them called *** used to beat us up at the smallest noise—he wanted us to be quiet every time—like a mortuary. We were not raped but just beaten many times and some people were killed.

*(Adult, M1)*

Yet, the experiences of violence were no less in official detention centers, some of which participants indicated were funded by the European Union and the United Nations.

*When we arrived, the other migrants who had been in the prison for a while started crying and screaming in Edo languages, ‘they have brought some people again oh’. In the prison, we only had one pair of panties… even when we were on our period. Whenever journalists came, the guards gave us juice to make it look like they were taking care of us. So many people had sores, people were dying of gunshot wounds, there was so much pain and sickness, the most others can do to help is carry you out and pour water on your head. Hunger is nothing again, we get used to it. One day a [business] man came and picked people, and carried them away to go and work, especially the people with babies. The soldiers at the prison collected money from UN endlessly.*

*(Adult, M1)*

The narratives of torture and neglect were just as rampant in the detention centers, and the labor exploitation element was common as well, when the guards colluded with locals who took the migrants away to work for them. This participant noted that she was in a center with UN signs and that there was funding available for their care, yet they were not taken care of at all, validating, once again, participants’ distrust of authorities.

- Connection houses (brothels) in the ghettos

A particular element of these respondents’ journey is that all participants ended up in a Libyan ghetto at some point during their journey, because the smugglers and traffickers were based there. The connection houses (where the connection men (smugglers) lived and housed or detained migrants) also operated as brothels and were often referred to as hell, horrible, and full of pain, because that was where many participants were raped and forced into prostitution for the first time. One participant was forced to undergo the juju ritual in Libya, which is rare, as the oath is usually carried out before commencement of the journey in Nigeria.

I was very surprised because that was not the plan or the agreement, but I had no choice, because I didn’t know anyone there and I had nowhere to go. That was when they forced me to swear, they took my nails and hair and forced her to drink some strange things. After that, they said I should work [prostitution] and I refused. So, they first threatened me, then *** [smuggler] and the Asma boys beat me and then they slept with me… I eventually joined the girls and began the ashawo [prostitution] work in Libya.

*(Adult, M1)*

Here, an alliance is seen between the smugglers/traffickers and the local militia, referred to as ‘asma boys’, who were brought in to enforce the will of the trafficker, showing her that her only option was to comply. In situations where the participants were forced into prostitution in Libya, the clients were often fellow Nigerians.

*When we got to Libya, they took me to the ghetto where my Boga [smuggler] lives and he put me in a small room there. He raped me, and also forced me to sleep with his [Nigerian] friends. So as soon as he had visitors, he would tell me to go into my room and wait for them, the friends would then eat and come to the room to have sex with me. Most of the time, they gave me small money when they finished. I always cried but that did not stop them… One day, I heard people running in the house and screaming that Asma boys were on the way so everyone ran away, but I was so sick that I could only crawl to try and escape. Then the asma boys (at least 5) came, they stole all the valuables in the house, then one by one they had sex with me, and they took me away with them. They raped me again, and eventually just dumped me in the desert to die. I really thought I would die this time, but again, I didn’t die… I got pregnant.*

*(Minor, M1)*

This participant was treated as a commodity by the smuggler who, while lying to her madam that he could not send her to Italy because it was not safe, ‘gave’ her to his friends to be used for their pleasure. This brutality that she experienced left her wanting to die and, during the first two measurement moments of our study, she was still unsure about whether she wanted to live or not.

Another participant mentioned being punished worse because of her nationality and race.

*In Libya too, the Arabu people just beat you anyhow. Once they see you are black and if they hear that you are from Nigeria, it is even worse! They will beat you, rape you, shoot the boys and nothing will happen!*

*(Minor, M1)*

This sentiment was shared by several of the participants who indicated that they were treated worse once the guards or their captors found out they were Nigerian. They also brought in the element of race and how Black people were treated worse than other migrants.

#### 3.1.3. Experiences of Violence: Italy

For eighteen of the participants, these violent encounters continued after their arrival in Italy. Although the violence was predominantly carried out by traffickers and local men, two participants described experiences of attempted rape and one experience of other physical brutality by male staff of the organizations whose care they were under.

- The streets

Interviewees who were forced into street prostitution after arrival in Italy described their experiences on the streets as unsafe and often terrifying.

*The streets were scary and starting to get cold at night at one point, and so many crazy customers. Some smelt bad, some with big stomach and strange things on their bodies, it was just horrible. They beat you, choke you, rape you and try to have sex without using a condom. One night I got into a fight with a customer who did not want to pay, and I refused to let him go until somebody called the police.*

*(Adult, M1)*

The participants were also attacked by pretend clients and exposed to danger by those refusing to use condoms, creating health risks for them and the possibility of infections and diseases.

*Aunty, street work is hell oh, the things I saw in that place. Customers beat me, some guys [Italian] would drive by and throw rubbish on me and the other girls, spit on us and even try to force them inside the car. I was raped [gangraped] by some guys who pretended to be customers and sometimes they won’t even use a condom. I am just thankful that I am alive.*

*(Minor, M1)*

That was when I started working on the streets which was hell. I saw people suffering, being beaten, raped by customers, I don’t even want to talk… The customers make you do horrible things. One man wanted me to *** [describes a sexual act], so I refused, and thank God he was not strong enough to force me, because I was struggling with him until I was able to run away. That is even the easy one. Some customers just come to kill you. I should not be alive today when I think about all of the things that I experienced.

*(Adult, M1)*

There were also instances where the participants were attacked by locals or passers-by.

So, I was hustling in *** [name of city]. It was at that time that those bad boys threw a bottle at me and hit me. I don’t know why, I stood by the roadside. (R: were they black boys?) No, they were white, and they threw a bottle at me. I was injured so the police came and called the ambulance which took me to the hospital.

*(Minor, M1)*

- Traffickers’ homes/brothels

Some participants were locked up by the traffickers after arrival in Italy to prevent escape and forced into prostitution in the traffickers’ homes.

*After the four days that she locked me up, my madam brought a man to the house and said that the man will use me, because he has already paid for sex with me, but I refused, and the madam told me that she had to look for her money and pay her… Madam started bringing Italian men home to have sex with me… Then later took me to the roadside to work with her there.*

*(Adult, M1)*

*When I got there [madam’s house], I was not allowed to leave the place. They told me that I would have to do street work and pay back, but since the madam could not trust me not to run away yet, for the first one month, they were bringing customers to me in the house, and I was not allowed to go out. I would cry every time they had sex with me, because that was not what they told me that I would come and do here, but I had no choice.*

*(Adult, M1)*

In these cases, the madam only let the participants work on the streets after they had been raped and ‘broken’ by the clients and she was sure they would not attempt to escape. These experiences of violence in the hands of clients and the madam may also evoke a loss of faith and trust in the system that they assumed would be better than the one they left behind. It also shows the dangerous positions that they were put in, so that they could earn money to repay the cost of the trip that already involved so much violence.

Other participants endured both sexual violence and physical brutality at the hands of the traffickers’ partners.

*When I was with the madam, her husband would beat me for every small thing that he said I did wrong, beat me on the bottom and on the breast and tried to rape me many times until I ran away.*

*(Minor, M1)*

The husband, he beat all of us. Already he called some of his friends, they were five men plus him which is six, and they all beat us, and they slept with us, and he killed one of our friends. He stabbed her with a bottle because he was drunk… We were all naked, *** was in front of him, he was standing there. So, he just got angry and broke the bottle and stabbed her, the girl fell down. When she fell down, he called us to wear her clothes for her and they took her out.

*(Minor, M1)*

While narrating this, the participant broke down, which illustrates the traumatizing effects these experiences must have on the participants.

- NGO centers/housing

Some interviewees also spoke about violence happening in reception centers.

*One time, I was very angry and went to the office to take the store key [without permission] and get food for myself and the man [NGO staff] told me to return the rice. I told him that I was pregnant and needed to cook and eat, so I would not return it, but he grabbed my neck and started choking me. I fought back and bit him, then he started squeezing my stomach and told me that he would kill me and my baby, and nothing would happen because I am just a Nigerian prostitute.*

*(Minor, M2)*

Another participant, also a minor, experienced attempted rape in her center.

*The new camp is ok, they have adults and minors there and the people working there are nice. One of the staff there tried to have sex with me, but I refused, and I warned him that if he tries again, I will break his head… He has not disturbed me again since that time, but he is always looking at me.*

*(Minor, M1)*

Another form of violence is that which is carried out by the girls themselves. Two participants described a situation in their shelter where a fellow migrant who was seven months pregnant had carried out an abortion on herself with the assistance of another girl. The two participants were horrified and shaken by what they had seen—the lifeless fetus. This happened in a ‘safe home’.

*Aunty, I saw them removing the baby. I quickly closed my eyes, so I won’t see but it was too late. The baby was complete oh, just dead, so sad.*

*(Adult, M2)*

During the study, many participants shared the different measures that they took to prevent pregnancy, such as douching, inserting herbs and certain capsules into their vaginas, and drinking homemade potions.

### 3.2. Impact on Participants’ Physical and Mental Health

Another key finding is how the participants’ experiences of violence continued to impact their physical and psychological wellbeing.

They expressed how their experiences of violence still caused them physical pain.

*When I think about it [the experiences of sexual violence], I feel chest pains.*

*(Minor, M2)*

*I often have trouble breathing and cannot take deep breaths. I have a lot of pain in my eyes and my legs shake [she described leg spasms].*

*(Adult, M1)*

*I went to the hospital because I have been unable to sleep because I have waist pain since we started the journey. *

*(Minor, M1)*

These experiences and their aftermath evoked different emotional reactions among the participants, with anger and fits of rage being amongst the most prevalent.

*I beat my child [conceived through rape] because sometimes, I see something in her, or one look on her face that I don’t know and I think is from her father, I feel very bad. *

*(Adult, M2)*

*Someone can do something very small to annoy me and I will just explode, and you will be wondering that is it because of this small thing that I am angry like this. I also drink alcohol sometimes, to cool my head. Ask the girls, they know me and my temper.*

*(Adult, M2)*

There were also examples of the impact of these violent experiences on their psychological wellbeing:

*Sometimes I can’t even eat when I think about it. I sometimes feel dizzy. Something has changed in my brain since then [the experiences of sexual violence]. I don’t reason well. Sometimes I just panic and then try to calm myself down.*

*(Adult, M1)*

*I forget things a lot. So much so, that I now set alarms on my phone so that I can remember… what I need to do, … where things are, like not leaving the iron on with children in the house where I lived.*

*(Adult, M2)*

These experiences also leave many of the participants fearful and feeling tormented, especially when they are alone.

*I experience that fear mostly if I’m alone, like if nobody is with me, I always experience it. I even called my sister in Nigeria and explained to her that sometimes my heart will just start beating fast like something bad is about to happen.*

*(Minor, M1)*

*If I close my eyes, I see horrible things that make me fear or I feel as if somebody is in the room, but I cannot see them. Sometimes, I will see a shadow but there is nobody else in the room, so who is the owner of the shadow?*

*(Adult, M2)*

*I no have roommate now. Hmm, I don’t like that… I don’t like to stay alone. Because at night, I am afraid. Sometimes even if I want to go to bed, I’ll stay in parlor [living room] until the sleep catches me [becoming extremely sleepy]... Without that, I can’t go to bed. I’ll just stay in the parlor. Sometimes I stay parlor till 5 am.*

*(Adult, M3)*

The violence that these women and girls experienced during their migration trajectories impacted them in multiple ways, forcing them to find ways to cope with them for survival.

### 3.3. Survival Strategies

This section looks at the different survival strategies that the participants adopted during their migratory journeys and how those strategies seemed to be the only way out in several instances.

Despite the violence and traumatic experiences that most of these teenagers and young women endured, they found ways, which were often difficult and, at times, just as traumatizing to stay alive and survive the extremely dangerous journeys that they found themselves on.

*In the desert, the Arab boys came at night to pick girls to sleep with, so I dressed like a boy to escape them, so that they will not force me to sleep with them.*

*(Adult, M1)*

This participant was able to escape sexual assault by deceiving the smugglers. She told this story with pride because amid the helplessness, she found a way to help herself. Although, she was beaten alongside the boys, she escaped the sexual violence that the girls in her group were subjected to.

Several participants ended up ‘dating’ or ‘starting a relationship’ with their captors and other men around them in Libya for the purpose of self-preservation, even though that was torturous as well.

People think I’m crazy? They think I want to f*** that wicked man [smuggler & trafficker] that tear my body with his big thing? I had no choice now. It was the only way to survive and get food for myself and my girlfriends in Libya… They beat and f*** my friends so badly that they had no energy to work or see customers, so they depended on me.

*(Minor, M1)*

Other participants described how they were able to escape Libya by being with men who could help and protect them.

*Erm, yes, I was made to work in Libya. That was, to me, that was how I made my way here. For me working in Libya, that was how I was able to come in interaction with a guy that helped me, a Malian guy that helped me escape.*

*(Adult, M1)*

*So, I was with the father of my baby and … I was there with him, but you know how it is, it’s not like you went there for love, but you are just like protecting yourself, you need shelter, a place that you can relax your soul. You know how it is in Libya, (sighs) Libya is not just a comfortable place for somebody to stay.*

*(Adult, M1)*

Both participants entered their relationships to escape Libya and be safe. They realized that traveling alone in Libya was not possible as they could fall into the hands of traffickers and militia, and quickly realized that the protection of a man would help their journey be smoother. This decision worked for both, and they were able to arrive in Italy. The participants also used similar strategies after arrival in Italy and living in NGO shelters, while waiting for their legal documentation to be processed and unable to work.

*There was a guy there that liked me, an Italian guy working at the shelter, and I slept with him a few times, but we were not together. Sleeping with him was good for me though because he made sure that I got the food and provisions that I wanted from the [shelter] store. *

*(Minor, M2)*

Others found illicit ways to acquire the money that they needed when they were desperate.

*I hustle… I do internet things to make money… chat with guys online, collect their money and disappear. *

*(Minor, M3)*

Lastly, we observed an overly sexualized atmosphere in certain shelters, where every comment, joke, or question was linked to a sexual act, connotation, or behavior (e.g., lap dancing, twerking, and even chasing a male member of staff with the determination to see his genitalia because they wanted to know what he was ‘working with’). This might refer to the lasting consequences of the participants’ experiences of sexual violence and abuse but can also be seen as a way of trying to cope with the past experiences of sexual violence.

## 4. Discussion

During this study, the first author’s understanding of the participants’ languages and nuances, being Nigerian herself, allowed for a deeper understanding of phrases, non-verbal expressions, etc. However, some of those expressions are extremely difficult to translate into English in the exact way they were expressed, although this was carried out as closely as possible.

Another point to reflect on is how the relationships with the participants, which were built over time during the longitudinal study, may have impacted the study. We believe that the longitudinal aspect better endeared the researcher to the participants as they mentioned that it was nice to have someone who cared enough about their experiences and stories to come back and listen. It also allowed for deeper trust to be built between the researcher and participants over time.

This study set out to document the narratives of Nigerian teenagers and women who were trafficked into Italy through the central Mediterranean route, in particular their experiences of violence and physical brutality along the way at the hands of different actors in the transit countries. It also showed the health impact of these experiences and the different coping strategies and survival mechanisms adopted by the participants, including hiding, finding ways to escape their captors, and entering relationships (which were sometimes purely sexual) with men for protection.

Our participants were very vocal about the myriad of violence that migrants traveling through the central Mediterranean route experienced, and they often felt some relief and a sense of comradery after finding out that others travelled along the same route that they did and were, for example, imprisoned in the same center that they were in. These narratives of violence along the way from Nigeria to Italy via the Niger–Libya route are consistent with reports and documentation of transit migrants’ experiences in Niger and Libya [45], and clearly question the consequences of the political choice to externalize European borders in the context of a broader European migration policy. Both the Republic of Niger and Libya are countries that the European Union has entered into agreements with, such as ‘The EUCAP Sahel Niger’ and the ‘Italy-Libya accord’, with the aim to curb migratory flows from Africa into Europe [46]. The increasingly central role of smugglers and traffickers in migrants’ trajectories, the shocking testimonies of migrants in Libyan slave markets, where Black migrants were being sold and bought by Libyans [47], together with the role of diverse detention systems in several transit countries (of which the Libyan detention centers are notoriously brutal) are just some of the consequences of these agreements, thereby, strongly increasing migrants’ vulnerability to experiencing a range of traumatic experiences, including repeated sexual violence, and evoking severe mental and physical health problems.

However, for many, the experiences of violence do not end after arrival in Italy, as they are forced into prostitution and beaten by clients, pretend clients, and traffickers alike. A recent study [48] among Nigerian women working in prostitution in the Brussels Red-Light District (many of whom were undocumented and arrived in Europe via the central Mediterranean route as well) also found that one of the women’s biggest concerns was safety. This was because of experienced violence at the hands of clients and pretend clients who raped them, attempted to do so, robbed, or attacked them, and violence perpetuated by locals who attacked them because they did not want them there. For several of our interviewees, sexual violence continued even after leaving the streets and the traffickers’ networks, while residing in care structures. The participants still experienced violence in different forms after arrival in safe houses and NGO homes. Two participants, both of whom were under the age of 18, were almost raped by caregivers whose job was to ensure their safety. Having a clear security protocol for the vetting and hiring of staff is crucial, including staff’s follow-up and the possibility for residents to safely narrate about these experiences to the care structure’s management without their safety being jeopardized. In one of the excerpts, the participant was told by her attacker (also one of her caregivers) that her life was worth nothing since she was merely ‘a Nigerian prostitute’, using a demeaning term and validating the claim that the predicaments of violence are exacerbated by socio-cultural factors, such as particular perceptions, values, and norms of certain groups [49].

The violence experienced by our participants during their migration trajectories is similar to that described by other migrants in transit while traveling irregularly [8], and after arrival in the destination country [12]. The experiences also show how these experiences of violence impact them physically, emotionally, and psychologically, and that the experiences of violence do not end after arrival in the destination country. These factors are important for caregivers, practitioners, and other involved stakeholders to know, as they show the impact of violence on these women and girls’ wellbeing and highlight the importance of appropriate and relevant policies and support systems.

## 5. Conclusions

The participants’ lived experiences and perspectives were crucial, as they highlight the extreme experiences they encountered as victims of trafficking during their migration trajectories. The re-victimization that is so apparent in our participants’ narratives is known to lead to serious mental health consequences, but also to put pressure on future exposure to (sexual) traumatic experiences and may negatively affect interpersonal relationships [50]. This does not only necessitate the urgency to decrease the possible traumatic exposure of migrants along their trajectories, and after arrival in Europe, but also calls out for a much greater emphasis on the integration of psychosocial and mental health programs in care structures, including, of course, diminishing the risk of being retraumatized in (safe) shelters by staff and other residents. It is important to note that the survival strategies that these women and girls are forced to adopt may be just as traumatizing as some of their experiences of violence. Therefore, it is crucial that caregivers and service providers consider their effects and impact while designing programs and services for the women and girls.

In conclusion, our study shows that, while there may be many shared experiences among irregular migrants and other victims of trafficking, there is an additional layer of violence experienced by victims of trafficking for sexual exploitation who are trafficked through irregular migration routes. Our research shows that multiple experiences of violence happen throughout their migration trajectories and that the violence continues after arrival in the country of destination, even in safe houses.

## Data Availability

The data that support the findings of this study are available from Ghent University, but restrictions apply to the availability of these data, which were used under license for the current study, and so are not publicly available.

## References

[1] International Labour Organization (2022). Global Estimates of Modern Slavery: Forced Labour and Forced Marriage; International Labour Organization. https://www.ilo.org/wcmsp5/groups/public/---ed_norm/---ipec/documents/publication/wcms_854733.pdf.

[2] United Nations Office on Drugs and Crime (2022). Global Report on Trafficking in Persons; United Nations Office on Drugs and Crime. https://www.unodc.org/documents/data-and-analysis/glotip/2022/GLOTiP_2022_web.pdf.

[3] European Asylum Support Office (EASO) Country of Origin Information Report; European Asylum Support Office. https://coi.easo.europa.eu/administration/easo/PLib/BZ0415678ENN.pdf.

[4] European Commission (2020). Report from the Commission to the European Parliament and the Council. Third Report on the Progress Made in the Fight against Trafficking in Human Beings (2020) as Required under Article 20 of Directive 2011/36/Eu on Preventing and Combating Trafficking in Human Beings and Protecting Its Victims; European Commission. https://ec.europa.eu/anti-trafficking/third-report-progress-made-fight-against-trafficking-human-beings_en.

[5] InfoMigrants (2019). Gender-Based Violence Top Reason Nigerian Women Leave. https://www.infomigrants.net/fr/post/16521/genderbased-violence-top-reason-nigerian-women-leave-report.

[6] Krug E.G., Dahlberg L., Mercy J.A., Zwi A., Lozano R. (2002). World Report on Violence and Health.

[7] United Nations Office on Drugs and Crime (2011). Trafficking in Persons to Europe for Sexual Exploitation. Trends Organ. Crime.

[8] Belanteri R.A., Hinderaker S.G., Wilkinson E., Episkopou M., Timire C., De Plecker E., Mabhala M., Takarinda K.C., Van den Bergh R. (2020). Sexual Violence against Migrants and Asylum Seekers. The Experience of the Msf Clinic on Lesvos Island, Greece. PLoS ONE.

[9] De Schrijver L., Vander Beken T., Krahé B., Keygnaert I. (2018). Prevalence of Sexual Violence in Migrants, Applicants for International Protection, and Refugees in Europe: A Critical Interpretive Synthesis of the Evidence. Int. J. Environ. Res. Public Health.

[10] Keygnaert I., Dialmy A., Manco A., Keygnaert J., Vettenburg N., Roelens K., Temmerman M. (2014). Sexual Violence and Sub-Saharan Migrants in Morocco: A Community-Based Participatory Assessment Using Respondent Driven Sampling. Glob. Health.

[11] Roupetz S., Garbern S., Michael S., Bergquist H., Glaesmer H., Bartels S.A. (2020). Continuum of Sexual and Gender-Based Violence Risks among Syrian Refugee Women and Girls in Lebanon. BMC Women’s Health.

[12] Freedman J. (2016). Sexual and Gender-Based Violence against Refugee Women: A Hidden Aspect of the Refugee “Crisis”. Reprod. Health Matters.

[13] Vu A., Adam A., Wirtz A., Pham K., Rubenstein L., Glass N., Beyrer C., Singh S. (2014). The Prevalence of Sexual Violence among Female Refugees in Complex Humanitarian Emergencies: A Systematic Review and Meta-Analysis. PLoS Curr..

[14] Johnson K., Scott J., Rughita B., Kisielewski M., Asher J., Ong R., Lawry L. (2010). Association of sexual violence and human rights violations with physical and mental health in territories of the Eastern Democratic Republic of the Congo. JAMA.

[15] Verelst A. (2014). The Psychosocial Well-Being of Adolescent Victims of Sexual Violence in Eastern Congo. Ph.D. Thesis.

[16] Babalola S.O. (2014). Dimensions and Correlates of Negative Attitudes toward Female Survivors of Sexual Violence in Eastern Drc. J. Interpers. Violence.

[17] Boakye K.E. (2009). Attitudes toward Rape and Victims of Rape: A Test of the Feminist Theory in Ghana. J. Interpers. Violence.

[18] Peltola L. (2018). Rape and Sexual Violence Used as a Weapon of War and Genocide. Bachelor Thesis.

[19] Munala J. (2007). Challenging Liberian Attitudes towards Violence against Women. Forced Migr. Rev..

[20] Kuhlken J., Rittner C., Roth J.K. (2012). Weapon of Sadness: Economic and Ethical Dimensions of Rape as an Instrument of War.

[21] Gaventa J. (1980). Power and Powerlessness: Quiescence and Rebellion in an Appalachian Valley.

[22] Bosi L., Dochartaigh N., Pisoui D. (2015). Political Violence in Context: Time, Space and Milieu.

[23] Esposito F., Quinto C.R., De Masi F., Gargano O., Costa P.A. (2016). Voices of Nigerian Women Survivors of Trafficking Held in Italian Centres for Identification and Expulsion. Int. Migr..

[24] Esposito F., Ornelas J., Scirocchi S., Arcidiacono C. (2019). Voices from the Inside: Lived Experiences of Women Confined in a Detention Center. Signs J. Women Cult. Soc..

[25] Aghatise E. (2005). Women Trafficking from West Africa to Europe: Cultural Dimensions and Strategies. Mozaik.

[26] Tessitore F., Margherita G. (2021). Female Nigerian Asylum Seekers in Italy: An Exploration of Gender Identity Dimensions through an Interpretative Phenomenological Analysis. Health Care Women Int..

[27] Tessitore F., Gallo M., Cozzolino M., Margherita G. (2022). The Frame of Nigerian Sex Trafficking between Internal and External Usurpers: A Qualitative Research through the Gaze of the Female Nigerian Cultural Mediators. Int. J. Appl. Psychoanal. Stud..

[28] Morales-Rodríguez F.M., Pérez-Mármol J.M. (2019). The Role of Anxiety, Coping Strategies, and Emotional Intelligence on General Perceived Self-Efficacy in University Students. Front. Psychol..

[29] Schapendonk J. (2018). Navigating the migration industry: Migrants moving through an African-European web of facilitation/control. J. Ethn. Migr. Stud..

[30] Uzureau O., Lietaert I., Senovilla Hernandez D., Derluyn I. (2022). Multi-Layered Mobilities: Unaccompanied Minors’ Trajectories, Decision Making and Mobility after Arrival in Italy. Dissertation Thesis.

[31] Behrendt M., Pfeiffer E., Devlieger I., Adeyinka S., Rota M., Uzureau O., Lietaert I., Derluyn I. (2022). The Impact of Daily Stressors on Unaccompanied Young Refugees’ Mental Health: A Longitudinal Study. Am. J. Orthopsychiatry.

[32] Orsini G., Rota M., Uzureau O., Behrendt M., Adeyinka S., Lietaert I., Derluyn I. (2022). Loops of Violence(S) within Europe’s Governance of Migration in Libya, Italy, Greece, and Belgium. Politics Gov..

[33] Pfeiffer E., Behrendt M., Adeyinka S., Devlieger I., Rota M., Uzureau O., Verhaeghe F., Lietaert I., Derluyn I. (2022). Traumatic Events, Daily Stressors and Posttraumatic Stress in Unaccompanied Young Refugees during Their Flight: A Longitudinal Cross-Country Study. Child Adolesc. Psychiatry Ment. Health.

[34] Braun V., Clarke V. (2006). Using thematic analysis in psychology. Qual. Res. Psychol..

[35] Todres L. (2007). Embodies Enquiry: Phenomenological Touchstones for Research, Psychotherapy and Spirituality.

[36] Stenner P. (2012). Inventive Methods: The Happening of the Social—Pattern.

[37] Saldaña J. (2016). The Coding Manual for Qualitative Researchers.

[38] Aikpitanyi I. (2011). 500 Storie Vere sulla Tratta delle Ragazze Africane in Italia.

[39] Babatunde A.O. (2014). Human Trafficking and Transnational Organized Crime: Implications for Security in Nigeria. Peace Res..

[40] Carling J. (2006). Migration, Human Smuggling and Trafficking from Nigeria to Europe; International Organization for Migration (IOM). https://publications.iom.int/books/mrs-ndeg23-migration-human-smuggling-and-trafficking-nigeria-europe.

[41] Baarda C.S. (2016). Human Trafficking for Sexual Exploitation from Nigeria into Western Europe: The Role of Voodoo Rituals in the Functioning of a Criminal Network. Eur. J. Criminol..

[42] Nagle L., Owasanoye B. (2016). Fearing the Dark: The Use of Witchcraft to Control Human Trafficking Victims and Sustain Vulnerability. Southwest. Law Review..

[43] Adeyinka S., Lietaert I., Derluyn I. (2022). The Role of Juju Rituals in Human Trafficking of Nigerians: Its Use as a Tool of Enslavement But Also One of Escape.

[44] McMahon S., Sigona N. (2021). Death and Migration: Migrant Journeys and the Governance of Migration during Europe’s “Migration Crisis”. Int. Migr. Rev..

[45] Amnesty International (2020). Libya: New Evidence Shows Refugees and Migrants Trapped in Horrific Cycle of Abuses. News Release. https://www.amnesty.org/en/latest/news/2020/09/libya-new-evidence-shows-refugees-and-migrants-trapped-in-horrific-cycle-of-abuses/.

[46] Bøås M. (2021). Eu Migration Management in the Sahel: Unintended Consequences on the Ground in Niger?. Third World Q..

[47] United Nations African Migrants Reportedly Being Sold in ‘Slave Markets’ in Libya, Un Agency Warns; United Nations, 11 April 2017. https://refugeesmigrants.un.org/african-migrants-reportedly-being-sold-‘slave-markets’-libya-un-agency-warns.

[48] Adeyinka S., Samyn S., Zemni S., Derluyn I. (2021). Nigerian and Ghanaian Women Working in the Brussels Red-Light District.

[49] Mwenyango H. (2021). Gendered Dimensions of Health in Refugee Situations: An Examination of Sexual and Gender-Based Violence Faced by Refugee Women in Nakivale Refugee Settlement, Uganda. Int. Soc. Work..

[50] Classen C.C., Palesh O.G., Aggarwal R. (2005). Sexual Revictimization: A Review of the Empirical Literature. Trauma Violence Abus..

